# Effectiveness of resistance strength training in children and adolescents with ≥30% total body surface area: A systematic review

**DOI:** 10.4102/sajp.v72i1.303

**Published:** 2016-06-29

**Authors:** Yolandi Brink, Heather Brooker, Emmari Carstens, Cary A. Gissing, Candice Langtree

**Affiliations:** 1 Department of Interdisciplinary Health Sciences, Stellenbosch University, South Africa

## Abstract

**Purpose:**

Children and adolescents with burn injuries are at risk of living with social, educational, physical and psychological impairments. The systematic review aimed to ascertain the effectiveness of resistance strength training on muscle strength and lean body mass (LBM) in children and adolescents with burn injuries.

**Method:**

Five databases were searched. Randomised controlled trials with an intervention defined as a supervised, individualised resistance exercise programme were sought. The outcomes included muscle strength and/or LBM. The PEDro scale was used to describe the methodological quality. Comparable data were combined using RevMan^©^.

**Results:**

Seven papers were included in the review with an average methodological appraisal score of 5.7/11. Comparable data were combined for muscle strength and LBM. The meta-analysis revealed no significant clinical difference between the exercise and standard care groups after 3 months of strength training for both muscle strength (*p* = 0.43) and LBM (*p* = 0.60).

**Conclusions:**

There is no conclusive evidence to support the benefit of strength training for children and adolescents with burns injuries in terms of muscle strength and LBM. However, it appears that isokinetic training might benefit children and adolescents with burns, but more studies investigating the effect of isokinetic training are required.

## Introduction

Burns contribute considerably to the global burden of injury among children (Scheven, Barker & Govindasamy 2012; WHO, media centre, fact sheet). Burns account for a quarter of a million deaths annually with the majority occurring in low- and middle-income countries (WHO 2010/2011). In Africa, burn injuries are the leading cause of accidental death in children (Scheven *et al*. 2012; WHO, media centre, fact sheet). Annually, about 1000 children with burns are treated at the largest children’s hospital in Africa (Van Niekerk *et al*. 2004). These burns were most commonly caused by exposure to boiling water, other hot liquids and flames, especially in the informal settlements where there is an increased usage of indoor fires and paraffin stoves (Van Niekerk *et al*. 2004).

Young children are at an increased risk of burns because of their curiosity to explore the environment in an immature manner (Van Niekerk, Rode & Laflamme 2004). Boys reportedly suffer from burns because of higher energy levels and mischievous behaviour (Van Niekerk *et al*. 2004). Burns in older girls are common because of their domestic roles in the household (WHO 2008). In children, head, neck and upper-body burns are most common (Van Niekerk *et al*. 2004). These impairments can markedly reduce their growth and function (Weedon & Potterton 2011).

Children and adolescents affected by burns often live with life-long social, educational, physical and psychological consequences (Rivlin & Faragher 2007; Weedon & Potterton 2011). They are vulnerable because of their maturing physical and psychosocial development (Russell *et al*. 2013; Stubbs *et al*. 2011; Toon *et al*. 2011). Because of the immense impact of burns injuries on their development, effective management strategies are crucial (Arceneaux & Meyer 2009).

Management of burns typically consists of medical treatment and physiotherapy and occupational therapy. The medical management consists of continuous debridement and excision of the necrotic tissue through surgical approaches, which also include full- and split-skin grafts for deep, partial, and full-thickness burns (Darwish 2011). Surgery encourages the healing process and optimal wound appearance and functionality. Physiotherapy management includes respiratory management, oedema control, stretching and strengthening exercises as well as splints to maintain the achieved range of movement (ROM) and for contracture prevention (Simons, King & Edgar 2003). Scar management includes compression and massaging techniques for the optimal formation of a functional scar. Aerobic exercise is an important element of physiotherapy management to improve exercise tolerance and cardiovascular endurance (Disseldorp *et al*. 2011). Physiotherapy is applied in various phases of burn rehabilitation because of the extensive physiological complications.

The inflammatory response after a burn injury triggers a hypermetabolic reaction. This is characterised by a hyperdynamic reaction with increased body temperature, oxygen and glucose consumption, CO_2_ production, glycogenolysis, proteolysis, lipolysis and futile substrate cycling (Jeschke *et al*. 2008). This hypermetabolic response continues up to 24 months post-burn, causing loss of lean body mass (LBM), bone density and muscle weakness (Atiyeh, Gunn & Dibo 2008; Esselman *et al*. 2006). Considering these cascade of events and the effect on muscle strength, it seems worthwhile for physiotherapists to consider resistance exercises as a strengthening modality for children and adolescents with burns.

The primary aim of resistance exercises is to improve muscle strength. Training close to the muscle’s force-generating capacity increases muscle tension, which initiates skeletal muscle growth, therefore affecting LBM (Grisbrook *et al*. 2013). Resistance training causes an acceleration of protein synthesis on cellular level by predominantly increasing the amount of contractile proteins, thus leading to muscle hypertrophy, which improves muscle size and force output (Grisbrook *et al*. 2013; Phillips *et al*. 1999).

Disseldorp *et al*. (2011) conducted a review to assess the effect of progressive resistance exercise (PRE) training on physical fitness of burn patients of all ages. The findings were synthesised descriptively, and they proposed that exercise training improves muscular strength, muscular and cardio-respiratory endurance, body composition and flexibility in children and adolescents. However, a meta-analysis was not conducted and sample sizes were small and not justified. Therefore, it remains unclear whether there is high level evidence to recommend PRE to clinicians. In addition, since the review by Disseldorp *et al*. (2011), more studies have been published.

The aim of this systematic review was to ascertain the effectiveness of the combination of an individualised, supervised strengthening programme (resistance exercises) with standard care to standard care alone, on muscle strength and LBM in children and adolescents with burn injuries >30% total body surface area (TBSA). The cut off of >30% TBSA was chosen as Baker *et al*. (2007) reported that an important long-term consequence of paediatric burns >30% TBSA was general muscle weakness affecting the function of young adults and the authors stressed that more emphasis should be placed on strengthening during the rehabilitation phase. Using meta-analytical analyses to increase statistical power of the treatment effect, clinical recommendations can then be based on the current evidence base.

## Methodology

### Search strategy

Five databases (CINAHL, Cochrane Library, PubMed, ScienceDirect and Scopus) were searched from inception to September 2015. The search was performed independently by two research groups (H.B., E.C., C.A.G., C.L. and Y.B., Q.L.), using the following key search terms: exercise, lean body mass, muscle strength, burns, thermal injuries, children and adolescents. The two research groups independently screened the titles and abstracts, thereafter potential full text papers were obtained and evaluated according to the inclusion and exclusion criteria.

### Inclusion criteria

Randomised controlled trials, published in English and available in full text, were included. There was no limitation in the range of publication date, and papers published up until September 2015 were included. Both male and female children and adolescents (6–18 years) with greater than 30% TBSA were the participants in the trials. The intervention was defined as a supervised, individualised (patient specific) resistance exercise programme of the upper and/or lower limbs in combination with standard care, which commenced within 1 year of the injury and continued for at least 12 weeks. The control group only received standard care consisting of burn wound medication, physiotherapy, wound care, psychological care, nutritional care and occupational therapy. The eligible papers must have reported on both muscle strength and/or LBM post-intervention. Muscle strength could be measured using a dynamometer; the 3 repetition maximum (3RM) technique or any other valid objective instrument. LBM could be measured with dual-energy X-ray absorptiometry (DEXA) or any other valid objective instrument.

### Exclusion criteria

Papers including participants with (1) leg amputation, (2) psychological disorders, (3) quadriplegia, (4) severe cognitive disorders, (5) developmental delay before hospitalisation, (6) neurological injury, (7) previously sustained significant hearing/vision loss and (8) post-burn injuries of more than 1 year were excluded from the review. The exclusion criteria include patients who would have been physically and/or mentally unable to complete the intervention programme over and above standard care procedures.

### Methodological appraisal

The PEDro scale was used to describe the methodological quality of the eligible studies. The PEDro score is based on the Delphi list and scales 11 items (Table 1). Items are scored as either present (1) or absent (0), and a score out of 11 is calculated by summation (De Morton 2009). Each paper was scored, and discrepancies were discussed in order to reach consensus. The scores from the PEDro scale are not indicative of the effectiveness or the clinical relevance of the studies (Verhagen *et al*. 1998).

**TABLE 1:** The methodological quality scoring of the eligible studies according to the PEDro scale.

Variables	Item 1	Item 2	Item 3	Item 4	Item 5	Item 6	Item 7	Item 8	Item 9	Item 10	Item 11
Cucuzzo *et al*. 2001	√	√	x	√	x	x	X	√	√	√	x
Suman *et al*. 2001	√	√	x	x	x	x	X	√	√	√	x
Przkora *et al*. 2007	√	√	x	√	x	x	x	√	√	√	x
Suman & Herndon 2007	√	√	x	x	x	x	x	√	√	x	x
Al-Mousawi *et al*. 2010	√	√	x	x	x	x	x	√	√	√	√
Ebid *et al*. 2014	√	√	√	√	x	x	√	√	√	√	√
Hardee *et al*. 2014	x	√	x	x	x	x	x	√	√	√	x

√ = adherence to the criteria; x = non-adherence to the criteria.

Item 1: specified eligibility criteria; Item 2: random allocation; Item 3: concealed allocation; Item 4: similarity at baseline; Item 5: subject blinding; Item 6: therapist blinding; Item 7: assessor blinding; Item 8: >85% follow-up for at least one outcome; Item 9: intention-to-treat analysis; Item 10: between-group statistical comparison for at least one key outcome, and Item 11: end point and variability measures for at least one key outcome.

### Data extraction and analysis

The adapted Joanna Briggs Institute Data Extraction Form was used to extract data from the papers under the following subheadings: citation, study type, participants, interventions (treatment and control group), outcome measures, results and the clinical status post-intervention and clinical implication thereof (Godfrey & Harrison 2012). When additional data were required, the authors were contacted in order to complete the data extraction process.

The comparable, homogenous data within individual studies, such as patient populations, interventions and outcome measures, were combined using the RevMan© Review Manager Software (RevMan© 2014). This meta-analysis allowed the interpretation of the effectiveness of the combination of a supervised strengthening exercise programme with standard care compared to standard care alone.

## Results

### Database search results

**FIGURE 1:**
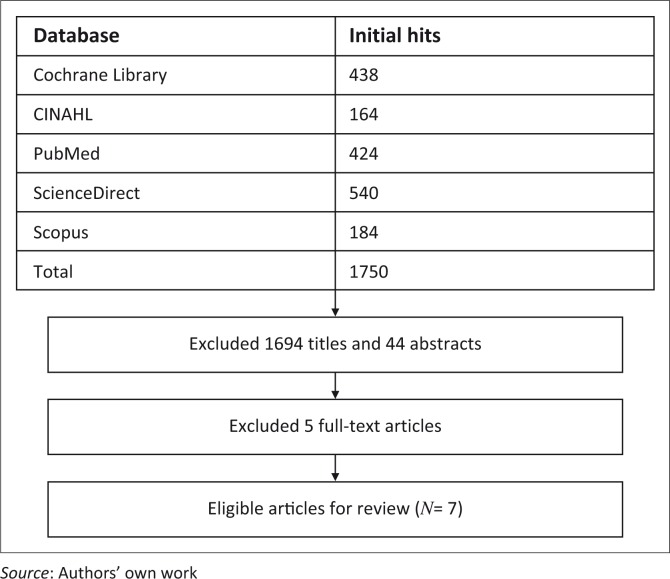
Results of the search strategy.

Seven eligible papers were included in the review (Al-Mousawi *et al*. 2010; Cucuzzo, Ferrando & Herndon 2001; Ebid, El-Shamy & Draz 2014; Hardee *et al*. 2014; Przkora, Herndon & Suman 2007; Suman *et al*. 2001; Suman & Herndon 2007) (Figure 1).

### Methodological appraisal

The average methodological appraisal score was 5.7 out of 11. Two studies scored 4 (Hardee *et al*. 2014; Suman & Herndon 2007), one study scored 5 (Suman *et al*. 2001), three studies scored 6 (Al-Mousawi *et al*. 2010; Cucuzzo *et al*. 2001; Przkora *et al*. 2007) and one study scored 9 (Ebid *et al*. 2014). None of the studies met the following two criteria on the PEDro scale: (5) blinding of subjects and (6) blinding of therapists (Table 1). Only two studies (Al-Mousawi *et al*. 2010; Ebid *et al*. 2014) provided point and variability measures in their published papers (Table 2). Furthermore, there was a difference in the mean values for muscle strength at baseline favouring the standard care group in three studies (Al-Mousawi *et al*. 2010; Suman *et al*. 2001; Suman & Herndon 2007). There was also a difference in the mean values for the baseline LBM assessments in two of the five studies, one favouring the exercise group and one the standard care group (Al-Mousawi *et al*. 2010; Suman & Herndon 2007).

**TABLE 2:** Means and SD of the baseline measurements for muscles strength and lean body mass in the exercise and standard care groups.

Variables	Muscle strength		Lean body mass	
	
	*Exc*	*SC*	*Exc*	*SC*
Cucuzzo *et al*. 2001	5.8 (0.7) kg	5.6 (1.1) kg	Not assessed	Not assessed
Suman *et al*. 2001	26.1 (17.4) Nm	34.3 (27.6) Nm	29.0 (15.7) kg	29.2 (12.1) kg
Przkora *et al*. 2007	35.4 (40.4) Nm	35.8 (31.9) Nm	31.2 (18.1) kg	31.5 (12.9) kg
Suman & Herndon 2007	31.3 (20.9) Nm	47.1 (27.6) Nm	36.9 (18.3) kg	34.6 (12.3) kg
Al-Mousawi *et al*. 2010	41.2 (38.7) Nm	57.8 (37.7) Nm	33.00 (14.9) kg	35.7 (16.0) kg
Ebid *et al*. 2014	47.06 (0.99) Nm	47.23 (0.97) Nm	Not assessed	Not assessed
Hardee *et al*. 2014	Not assessed	Not assessed	34.3 (NR)	34.8 (NR)

E*xc* = exercise group; *SC* = standard care group; NR = not reported.

### Study samples

The seven eligible studies varied in sample size, ranging from 19 to 47 participants. The mean age was similar across studies ranging from 9.2 to 13.7 years (the SD was not reported in all studies), although both exercise and standard care groups favoured more male than female participants (Table 3). Six studies included participants with ≥ 40% TBSA, and one study by Ebid *et al*. (2014) included participants with ≥ 36% TBSA. All the studies except one (Ebid *et al*. 2014) were conducted at the same institution in the United States.

**TABLE 3:** The sample size and gender distribution of each study.

Variable	Cucuzzo *et al*. 2001	Suman *et al*. 2001	Przkora *et al*. 2007	Suman & Hernson 2007	Al-Mousawi *et al*. 2010	Ebid *et al*. 2014	Hardee *et al*. 2014
Sample size							
*Exc*	11	19	11	11	11	16	24
*SC*	10	16	17	8	10	17	23
Male:Female						
*Exc*	8:3	16:3	13:4	9:2	9:2	10:6	20:4
*SC*	5:5	12:4	9:2	8:1	7:3	11:6	18:5
Age range for both groups (years)	5.9–19.9	7–17	7–17	7–18	7–17	10–15	N/R
Age (years)							
Mean (SD)							
*Exc*	11.9 (N/R)	10.5 (N/R)	10.9 (N/R)	11.9 (N/R)	12.2 (3.2)	13.46 (1.18)	13 (N/R)
*SC*	9.2 (N/R)	11.0 (N/R)	11.8 (N/R)	13.4 (N/R)	13.7 (3.6)	13.6 (1.12)	13 (N/R)

*Exc* = exercise group; *SC* = standard care group; N/R = not reported.

### Study interventions

All seven interventions were hospital-based individualised and supervised exercise programmes (Table 4). Six of the interventions were similar and involved PRE (Al-Mousawi *et al*. 2010; Cucuzzo *et al*. 2001; Hardee *et al*. 2014; Przkora *et al*. 2007; Suman *et al*. 2001; Suman & Herndon 2007). The intervention by Ebid *et al*. (2014) included isokinetic training. Five of the studies commenced with the intervention 6 months post-burn injury, whereas two studies (Ebid *et al*. 2014; Hardee *et al*. 2014) commenced after 1 month.

**TABLE 4:** Description of the interventions and standard care for the seven studies.

	Resistance exercise programme	Week 1	Weeks 2–6	Weeks 7–12	Weekly interval and duration	Additional training Weeks 1–12	Weekly interval and duration	Standard care
Cucuzzo *et al*. 2001	PRE	1–2 sets at low volume	50% of 3RM (4–10 reps)	70–85% of 3RM (8-15 reps)	3x/week for 60 min	General conditioning	3x/week for 60 min	Scar management; wound care
Suman *et al*. 2001	PRE	50–60% of 3RM	70–75% of 3RM (4–10 reps)	80–85% of 3RM (8–12 reps)	Not specified	Treadmill and cycle ergometer	3x/week for 20–40 min	Physiotherapy without exercise
Przkora *et al*. 2007	PRE	50–60% of 3RM	70–75% of 3RM (4–10 reps)	80–85% of 3RM (8–12 reps)	Not specified	Treadmill and cycle ergometer	5x/week for 20–40 min	ROM exercise; scar management; splinting; positioning
Suman & Herndon 2007	PRE	50–60% of 3RM	70–75% of 3RM (4–10 reps)	80–85% of 3RM (8–12 reps)	3x/week	Treadmill and cycle ergometer	3x/week for 20–40 min	Physiotherapy without exercise
Al-Mousawi *et al*. 2010	PRE	50–60% of 3RM	70–75% of 3RM (4–10 reps)	80–85% of 3RM (8–12 reps)	Not specified	Treadmill and cycle ergometer	3x/week for 30 min	ROM exercise; scar management; splinting; positioning
Ebid *et al*. 2014	Isokinetic exercises	50% of average peak torque (initial dose); 1–5 sets (10 reps/set)	6 sets (10 reps per set)	10 sets (10 reps per set)	3x/week	Home-based physiotherapy	Not specified	ROM exercise; scar management; splinting; positioning; daily walking
Hardee *et al*. 2014	PRE	Training of technique	50–60% of 3RM	80–85% of 3RM (8–12 reps)	Not specified	Treadmill and cycle ergometer	3–5x/week for 20–40 min	ROM exercise; scar management; splinting; positioning

PRE = progressive resistance exercises; ROM = range of movement; 3RM = 3 repetition maximum.

The interventions persisted for 12 weeks post-baseline assessment. The standard care, which was continued from hospital discharge and persisted for the duration of the intervention, included conventional physiotherapy and occupational therapy without individualisation or supervision of exercises. All standard care programmes were home-based except for Hardee *et al*. (2014), who implemented a hospital-based programme (Table 4).

### Study outcome measures

The outcome measures of interest were muscle strength and LBM. All studies assessed knee extensor strength of the dominant leg, using the Biodex System-3 Dynamometer (Al-Mousawi *et al*. 2010; Ebid *et al*. 2014; Hardee *et al*. 2014; Przkora *et al*. 2007; Suman & Herndon 2007), the Cybex Norm Dynamometer (Suman *et al*. 2001) or the 3RM (Cucuzzo *et al*. 2001). Cucuzzo *et al*. (2001) also assessed biceps, triceps, forearm and hamstring strength. LBM was assessed in five studies using DEXA with the QDR 4500A densitometry system (Al-Mousawi *et al*. 2010; Hardee *et al*. 2014; Przkora *et al*. 2007; Suman *et al*. 2001; Suman & Herndon 2007). Five studies performed baseline measurements at 6 months post-injury with post-intervention measurements at 9 months post-injury (12 weeks post-intervention) (Al-Mousawi *et al*. 2010; Cucuzzo *et al*. 2001; Przkora *et al*. 2007; Suman *et al*. 2001; Suman & Herndon 2007).

Two studies performed baseline measurements at 1 month post-injury with post-intervention measurements at 4 months post-injury (12 weeks post-intervention) (Ebid *et al*. 2014; Hardee *et al*. 2014). However, Hardee *et al*. (2014) only measured LBM at baseline and not muscle strength. Suman & Herndon (2007) also assessed both LBM and muscle strength at 1 year post-injury, 3 months post-cessation of the intervention, whereas Hardee *et al*. (2014) assessed LBM at 1 year post-injury, 8 months post-cessation of the intervention.

### Effect of a resistance exercise programme on muscle strength and LBM in children and adolescents with burns

Two studies could not be included in the meta-analysis because Cucuzzo *et al*. (2001) and Hardee *et al*. (2014) did not report the post-intervention point and variability measures (mean and SD) of muscle strength and LBM, respectively, and the authors did not respond to email communication.

## Muscle strength

Hardee *et al*. (2014) reported an insignificant mean difference in muscle strength between the exercise and standard care groups post-intervention (*p* = 0.08). Cucuzzo *et al*. (2001) and Ebid *et al*. (2014) reported significant within-group mean differences for both the exercise and standard care groups. Three studies (Al-Mousawi *et al*. 2010; Suman *et al*. 2001; Suman & Herndon 2007) have shown significant within-group mean differences for the exercise groups only (*p* <0.05).

**FIGURE 2:**
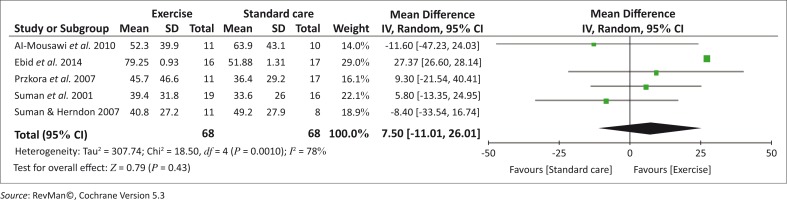
A forest plot to show the summary estimate of the average effect of resistance strengthening exercises on muscle strength at 3 months post-intervention.

The comparable data (means and SD) for the outcome muscle strength were combined from five studies (Al-Mousawi *et al*. 2010; Ebid *et al*. 2014; Przkora *et al*. 2007; Suman *et al*. 2001; Suman & Herndon 2007) and the meta-analysis revealed that there is no clinically significant difference between the exercise and standard care groups after 3 months of individualised supervised resistance strength training (*p* = 0.43). Heterogeneity in the summary effect of the combined studies was significantly (*p* = 0.001) high (78%) (Figure 2). This could be because of clinical differences between the studies in terms of the type of resistance training and the time interval post-burn injury when the intervention commenced. Ebid *et al*. (2014) included isokinetic resistance exercises and commenced with the intervention 1 month post-burn, whereas the other four studies implemented PREs as the intervention and commenced 6 months post-burn injury (Al-Mousawi *et al*. 2010; Przkora *et al*. 2007; Suman *et al*. 2001; Suman & Herndon 2007).

**FIGURE 3:**
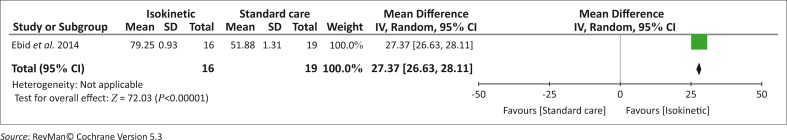
A forest plot to show the estimate of the effect of isokinetic training on muscle strength at 3 months post-intervention.

**FIGURE 4:**
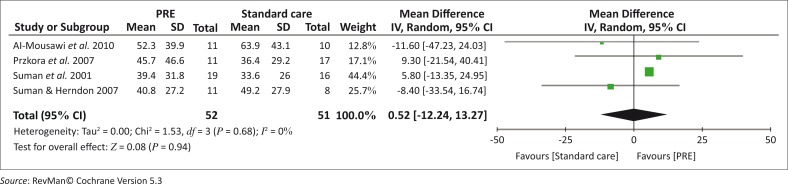
A forest plot to show the summary estimate of the average effect of progressive resistance exercise (PRE) on muscle strength at 3 months post-intervention.

Methodological differences between the studies could also explain the high level of heterogeneity since Ebid *et al*. (2014) scored 9 and the other four studies (Al-Mousawi *et al*. 2010; Przkora *et al*. 2007; Suman *et al*. 2001; Suman & Herndon 2007) scored between 4 and 6 out of 11 on the PEDro scale. Furthermore, Ebid *et al*. (2014), whose study had the greatest weighted percentage effect on the summary estimate, could also be seen as an outlier, thus increasing the heterogeneity. Therefore Ebid *et al*. (2014) and the other four studies (Al-Mousawi *et al*. 2010; Przkora *et al*. 2007; Suman *et al*. 2001; Suman & Herndon 2007) were analysed separately and the forest plots are presented in Figures 3 and 4.

The estimated treatment effect of isokinetic strength training showed a significant clinical effect (*p* <0.00001) favouring the exercise group compared to standard care alone. Although the combined effect of the studies implementing PRE indicated no heterogeneity between the studies, the summary estimate of the average effect of PRE on muscle strength revealed that there is no clinically significant difference between the exercise and the standard care groups (*p* = 0.94).

Suman and Herndon (2007) also reported a significant increase in mean percentage change for muscle strength in both the exercise (17.9%) and standard care (7.2%) groups at 1 year post-burn; however, neither increase were significant.

## Lean body mass

**FIGURE 5:**
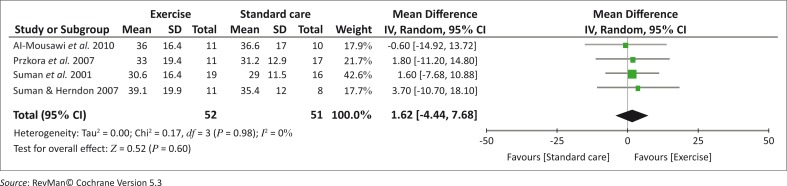
A forest plot to show the summary estimate of the average effect of strengthening exercises on LBM at 3 months post-intervention.

Four of the five studies(Al-Mousawi *et al*. 2010; Hardee *et al*. 2014; Suman *et al*. 2001; Suman & Herndon 2007) reported significant within-group mean differences for the exercise groups only (*p* <0.05). Similarly, a meta-analysis of the comparable LBM data from four studies (Al-Mousawi *et al*. 2010; Przkora *et al*. 2007; Suman *et al*. 2001; Suman & Herndon 2007) revealed that there is no clinically significant difference in LBM between the exercise and standard care groups after the 3-month intervention (*p* = 0.60). The heterogeneity was 0% indicating that the studies were homogenous with their confidence interval overlapping (Figure 5). Both Suman and Herndon (2007) and Hardee *et al*. (2014) reported significant increases in mean percentage change at 1 year post-burn in the exercise groups only (*p* <0.05).

## Discussion

This systematic review analysed the effect of a 12-week resistance strengthening exercise programme in addition to standard care compared to standard care alone, when managing children or adolescents with burns. The meta-analysis indicated no added benefit for muscle strength and LBM because of strength training when the intervention is implemented within 1–6 months post-injury.

The lack of a clinical difference in muscle strength post-intervention between the exercise and standard care groups could be because of the difference in baseline muscle strength values between the two groups (Table 2). The two studies (Ebid *et al*. 2014; Przkora *et al*. 2007) with comparable muscle strength at baseline show a clinical difference favouring the exercise groups; however, the two studies (Al-Mousawi *et al*. 2010; Suman & Herndon 2007) favouring the standard care group post-intervention also had stronger participants at baseline.

Although for LBM the groups were comparable at baseline, the summary effect of the intervention still showed no difference between the exercise and standard care groups following an individualised and supervised resistance strengthening exercise programme. Because the two studies assessed the long-term effects of the strengthening exercise programme on LBM and found persistent significant increases in LBM after cessation of the intervention, it could be that the treatment effect only becomes apparent in the long run and that longer follow-up periods need to be explored in future research. Furthermore, Ebid *et al*. (2012) conducted a trial among adults and reported a significant improvement in muscle strength and LBM for long-term burn patients and comparable non-injured adults following the same isokinetic training programme. This supports the potential benefit for long-term patients and implies that burn patients respond in the same way as non-injured controls (Ebid *et al*. 2012).

Small sample sizes and poor methodological quality (low PEDro scores) could also contribute to the inconclusive evidence that resistance strengthening exercises post-burn is effective in improving muscle strength and LBM. Blinding of subjects and therapists is challenging, as participation in the 12-week exercise programme compared to the standard care group would be evident. However, blinding of the assessors, concealed allocation and reporting measures of variability are possible, thus limiting the measurement bias. Including both children and adolescents in one group could also have influenced the results. A review by Schranz, Tomkinson and Olds (2013) found that age (as defined as younger and older than 12 years) has a significant effect on muscle strength following a resistance exercise strengthening programme, where the older adolescent responded more favourably to the intervention compared to the younger child. Future studies should investigate resistance strength training in children separately from adolescents.

Nevertheless, individual studies have reported significant within-group improvement in either or both LBM and muscle strength post-intervention, indicating that a resistance strengthening exercise programme could potentially be beneficial for children and adolescents with burns in improving LBM and muscle strength. Improvement in muscle strength is attributed to increased amounts of newly acquired actin and myosin proteins in the muscular contractile apparatus and an increased amount of contractile protein causes muscle hypertrophy, thus also leading to an increased total LBM. Although it appears that there is some merit in implementing supervised individualised isokinetic strength training for children and adolescents with burns 1 month post-burn injury, only one study by Ebid *et al*. (2014) implemented this strategy; thus, it remains inconclusive and more similar studies are required. Improved LBM and muscle strength might contribute to better quality of life and functional abilities, enabling children and adolescents to successfully reintegrate into their communities.

Future studies should focus on improving the methodological quality of the studies to confirm the treatment effect of a resistance strengthening exercise programme on muscle strength and LBM. Only one study (Ebid *et al*. 2012) was not conducted in the United States, and the other six studies were implemented at the same institution. This increases selection bias as only participants within a particular geographical area could potentially participant in these studies. Therefore, future research should be implemented internationally to be able to generalise findings and apply them globally to children and adolescents. Studies should lengthen the time period for data collection post-intervention to obtain an indication of the long-term effects of a resistance strengthening exercise programme on muscle strength and LBM.

## Conclusion

This review concludes that, at present, there is no substantial evidence to support the added benefit of a resistance strengthening exercise programme for children and adolescents with burns in terms of muscle strength and LBM. Because no deterioration of participants’ muscle strength and LBM was reported post-intervention, it appears that there is some merit in implementing supervised individualised isokinetic strength training for children and adolescents with burns; however, future research should pursue further investigation into the effectiveness of a resistance strengthening exercise programme on muscle strength and LBM.

## References

[CIT0001] Al-Mousawi A.M., Williams F.N., Mlcak R.P., Jeschke M.G., Herndon D.N. & Suman O.E., 2010, ‘Effects of exercise training on resting energy expenditure and lean mass during paediatric burn rehabilitation’, *Journal of Burn Care and Research* 31, 400–408. 10.1097/BCR.0b013e3181db5317 20354445 PMC3856323

[CIT0002] Arceneaux L.L. & Meyer W., 2009, ‘Treatment for common psychiatric conditions among children and adolescents during acute rehabilitation and reintegration phases of burn injury’, *International Review of Psychiatry* 21, 549–558. 10.3109/09540260903343984 19919208 PMC5201169

[CIT0003] Atiyeh B.S., Gunn S.W.A. & Dibo S.A., 2008, ‘Metabolic implications of severe burn injuries and their management: A systematic review of the literature’, *World Journal of Surgery* 32, 1857–1869. 10.1007/s00268-008-9587-8 18454355

[CIT0004] Baker C.P., Russell W.J., Meyer M.A. & Blakeney P., 2007, ‘Physical and psychologic rehabilitation outcomes for young adults burned as children’, *Archives of Physical Medicine and Rehabilitation* 88, S57–S64. 10.1016/j.apmr.2007.09.014 18036983

[CIT0005] Cucuzzo N.A., Ferrando A. & Herndon D.N., 2001, ‘The effects of exercise programming vs traditional outpatient therapy in the rehabilitation of severely burned children,’ *Journal of Burn Care and Research* 22, 214–220. 10.1097/00004630-200105000-00006 11403243

[CIT0006] Darwish A., 2011, ’Skin grafts – Indications, applications and current research’, in Spear M. (ed.), *Indication of skin grafts*, pp. 35–36, Intech Publisher, viewed 13 October 2015, from http://cdn.intechopen.com/pdfs/18926.pdf

[CIT0007] De Morton N.A., 2009, ‘The PEDro scale is a valid measure of the methodological quality of clinical trials: A demographic study’, *Australian Journal of Physiotherapy* 55, 129–133. 10.1016/S0004-9514(09)70043-1 19463084

[CIT0008] Disseldorp L.M., Nieuwenhuis M.K., Van Baar B.E. & Mouton L.J., 2011, ‘Physical fitness in people after burn injury: A systematic review’, *Archives of Physical Medicine and Rehabilitation* 92, 1501–1510. 10.1016/j.apmr.2011.03.025 21878221

[CIT0009] Ebid A.A., El-Shamy S.M. & Draz A.H., 2014, ‘Effect of isokinetic training on muscle strength, size and gait after healed pediatric burn: A randomized controlled trial’, *Burns* 40, 97–105. 10.1016/j.burns.2013.05.022 24074720

[CIT0010] Ebid A.A., Omar M.T.A. & Abd El Baky A.M., 2012, ‘Effect of 12-week isokinetic training on muscle strength in adult with healed thermal burn’, *Burns* 38, 61–68. 10.1016/j.burns.2011.05.007 22103985

[CIT0011] Esselman P.C., Thombs B.D., Magyar-Russell G. & Fauerbach J.A., 2006, ‘Burn rehabilitation: State of the science’, *American journal of Physical Medicine and Rehabilitation* 85, 383–413. 10.1097/01.phm.0000202095.51037.a3 16554686

[CIT0012] Godfrey C.M. & Harrison M.B., Version 3.0 June 27, 2012, *CAN-SYNTHESIZE is a quick reference resource to guide the use of the Joanna Briggs Institute methodology of synthesis**, Queen’s Joanna Briggs Collaboration, viewed 13 October 2015, from http://joannabriggs.org/assets/docs/jbc/operations/cansynthesise/CAN_SYNTHESISE_Appendices-V3.pdf

[CIT0013] Grisbrook T., Elliott C., Edgar D., Wallman K., Wood F. & Reid S., 2013, ‘Burn-injured adults with long term functional impairments demonstrate the same response to resistance training as uninjured controls’, *Burns* 39, 680–686. 10.1016/j.burns.2012.09.005 23021312

[CIT0014] Hardee J.P., Porter C., Sidossis L.S., Borsheim E., Carson J.A., Herndon D.N., et al, 2014, ‘Early rehabilitative exercise training in the recovery from pediatric burn’, *Medicine and Science in Sports and Exercise* 46, 1710–1716. 10.1249/MSS.0000000000000296 24824900 PMC4122649

[CIT0015] Jeschke M.G., Chinkes D.L., Finnerty C.C., Kulp G., Suman O.E., Norbuty W.B., Branski L.K., Gauglitz G.G., Mlcak R.P. & Herndon D.N., 2008, ‘Pathophysiologic response to severe burn injury’, *Annals of Surgery* 248, 387–401. 10.1097/sla.0b013e3181856241 18791359 PMC3905467

[CIT0016] Phillips S.M., Titon K.D., Ferrando A.A. & Wolfe R.R., 1999, ‘Resistance training reduces the acute exercise induced increase in muscle protein turnover’, *American Journal of Physiology, Endocrinolgy and Metabolism* 276, E118–E124. 10.1152/ajpendo.1999.276.1.E118 9886957

[CIT0017] Przkora R., Herndon D.N. & Suman O.E., 2007, ‘The effects of oxandrolone and exercise on muscle mass and function in children with severe burns’, *Paediatrics* 119, e109–e116. 10.1542/peds.2006-1548 PMC2367234 17130281

[CIT0018] RevMan© Cochrane Version 5.3, 2014, computer software, *Informatics and Knowledge Management Department*, viewed 13 October 2015, from http://tech.cochrane.org/revman

[CIT0019] Rivlin E. & Faragher E.B., 2007, ‘The psychological effects of sex, age at burn, stage of adolescence, intelligence, position and degree of burn in thermally injured adolescents: Part 2’, *Developmental Neurorehabilitation* 10, 173–182. 10.1080/17518420701309667 17687990

[CIT0020] Russell W., Robert R.S., Thomas C.R., Holzer C.E., Blakeney P. & Meyer W.J., 2013, ‘Self-perception of young adults who survived severe childhood burn injury’, *Journal of Burn Care Research*, 34, 394–402. 10.1097/BCR.0b013e3182700198 23202876 PMC3594050

[CIT0021] Scheven D., Barker P. & Govindasamy J., 2012, ‘Burns in rural Kwa-Zulu Natal: Epidemiology and the need for community health education’, *Burns* 38, 1224–1230. 10.1016/j.burns.2012.04.001 22698838

[CIT0022] Schranz N., Tomkinson G. & Olds T., 2013, ‘What is the effect of resistance training on the strength, body composition and psychosocial status of overweight and obese children and adolescents? A systematic review and meta-analysis’, *Sports Medicine* 43, 893–907. 10.1007/s40279-013-0062-9 23729196

[CIT0023] Simons M., King S. & Edgar D., 2003, ‘Occupational therapy and physiotherapy for the patient with burns: Principles and management guidelines’, *Journal of Burn Care & Research* 24, 323–335. 10.1097/01.BCR.0000086068.14402.C6 14501405

[CIT0024] Stubbs T.K., James L.E., Daugherty M., Epperson K., Barajaz K.A., Blakeney P., et al, ‘Psychosocial impact of childhood face burns: A multicentre, prospective, longitudinal study of 390 children and adolescents’, *Burns* 37, 387–394. 10.1016/j.burns.2010.12.013 21330061

[CIT0025] Suman O.E. & Herndon D.N., 2007, ‘Effect of cessation of a structured and supervised exercise conditioning program on lean body mass and muscle strength in severely burned children’, *Archives of Physical Medicine and Rehabilitation* 88, S24–S29. 10.1016/j.apmr.2007.09.002 18036977

[CIT0026] Suman O.E., Spies R.J., Celis M.M., Mlcak R.P. & Herndon D.N., 2001, ‘Effects of a 12-wk resistance exercise program on skeletal muscle strength in children with burn injuries’, *Journal of Applied Physiology* 91, 1168–1175. 11509512 10.1152/jappl.2001.91.3.1168

[CIT0027] Toon M.H., Maybauer D.M., Arceneaux L.L., Fraser J.F., Meyer W., Runge A., et al, 2011, ‘Children with burn injuries – Assessment of trauma, neglect, violence and abuse’, *Journal of Injury Violence Research* 3, 98–110. 10.5249/jivr.v3i2.91 21498973 PMC3134932

[CIT0028] Van Niekerk A., Du Toit N., Nowell M.J., Moore S. & Van As A.B., 2004, ‘Childhood burn injury: Epidemiological, management and emerging injury prevention studies. Crime, violence and injury prevention in South Africa: Developments and challenges’, *Medical Research Council*, 145–157, viewed 13 October 2015, from http://www.mrc.ac.za/crime/1streviewchapter9.pdf

[CIT0029] Van Niekerk A., Rode H. & Laflamme L., 2004, ‘Incidence and patterns of childhood burn injuries in the Western Cape, South Africa,’ *Burns*, 30, 341–347. 10.1016/j.burns.2003.12.014 15145192

[CIT0030] Verhagen A.P., de Vet H.C.W., de Brie R.A., Kessels A.G.H., Boers M., Bouter L., et al, 1998, ‘The Delphi list: A criteria list for quality assessment of randomised clinical trials for conducting systematic reviews developed by Delphi consensus’, *Journal of Clinical Epidemiology* 51, 1235–1241. 10.1016/S0895-4356(98)00131-0 10086815

[CIT0031] Weedon M. & Potterton J., 2011, ‘Socio-economic and clinical factors predictive of paediatric quality of life post burn’, *Burns* 37, 572–579. 10.1016/j.burns.2010.12.002 21251761

[CIT0032] World Health Organization, *Media centre, fact sheet*, viewed 13 October 2015, from http://www.who.int/mediacentre/factsheets/fs365/en/

[CIT0033] World Health Organization, *Violence, injuries and disability biennial 2010 / 2011 report*, viewed 13 October 2015, from http://apps.who.int/iris/bitstream/10665/75573/1/9789241504133_eng.pdf

[CIT0034] World Health Organization, *World report on child injury prevention 2008. Chapter 4 Burns*, viewed 13 October 2015, from http://www.who.int/violence_injury_prevention/child/injury/world_report/Burns.pdf?ua = 1

